# Right Ventricle Failure in Sepsis: A Case Report

**DOI:** 10.4021/cr12w

**Published:** 2011-01-20

**Authors:** Umashankar Lakshmanadoss, Bryana M Levitan, David H Hsi

**Affiliations:** a Division of Hospital Medicine, Johns Hopkins Bayview Medical Center, Johns Hopkins University, Baltimore, MD, USA; b Division of Cardiology, Unity Health System, Rochester, NY, USA

**Keywords:** Sepsis, RV dysfunction, Pulmonary hypertension, Central venous pressure, Fluid therapy

## Abstract

Sepsis could produce myocardial depression and typically it affects the left ventricle (LV). Sepsis could also affect right ventricle (RV), in addition to the interdependence with LV. RV pressure may be elevated secondary to pulmonary vasoconstriction, leading to RV dysfunction. Unlike LV, RV is poorly prepared to compensate for acute overload. Aggressive volume replacement may be vital to maintain RV function, but excess hydration can cause RV dilation, decreased LV diastolic filling, and reduced cardiac output. In patients having signs of inadequate cardiac output even after initial volume resuscitation, RV function should be assessed with echocardiogram. If RV dysfunction is noted, then fluid therapy should be guided by CVP measurements. If cardiac output increases with increasing CVP, maintaining higher filling pressures on the right side is indicated. On the other hand, increasing CVP with worsening of the cardiac output could worsen the RV dysfunction. In addition to the fluid management, treatment of other reversible causes like acidosis and hypoxia is also a key.

## Introduction

There is growing evidence that sepsis-associated cardiac dysfunction is not limited to the Left Ventricle (LV) but often involves the Right Ventricle (RV) as well. Normally, the RV has little trouble keeping up with the LV; however, in the presence of decreased contractility, increased afterload, or insufficient filling, which are three conditions commonly associated with sepsis, RV output may be unable to keep pace with increased LV output. Persistent RV dysfunction in sepsis has been associated with worse outcome. We report a case of systemic sepsis induced right ventricular (RV) failure, pulmonary hypertension and eventual improvement.

## Case Report

**Figure 1:**
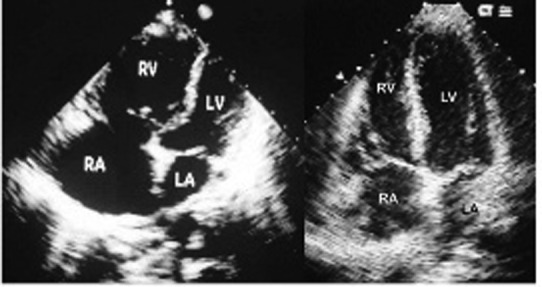
Echocardiogram in apical four chamber view. Left: Severe RV and RA dilatation. Right: Mild RV and RA dilatation in recovery.

Fifty-one-year-old Caucasian female was admitted to our hospital with complaints of fever and chills for three days and low back pain for two days. Past medical history included recent spinal abscess followed by implantation of spinal nerve stimulator, mitral valve prolapse and GERD. Her medications included omeprazole and acetaminophen as needed for chronic back pain. On physical examination, patient appeared sick. Vital signs: BP 80/50 mmHg, Pulse 120/min, respiratory rate 24/min. No skin lesions; neck was supple. Jugular veins were distended up to angle of the ear with prominent ‘a’ and ‘v’ waves. Her P2 was loud; No murmurs or gallop; lung fields were clear with normal vesicular breath sounds. Abdomen was soft and non tender. She had increased warmth and tenderness at the level of D10 - D12 vertebrae. Neurological examination was normal. Her labs revealed WBC count of 15,000 with bandaemia of 20%, lactic acid of 45 mgs%. Her EKG revealed sinus tachycardia. Serial troponin levels were normal. She was treated with broad spectrum antibiotics, intravenous fluids and dopamine as infusion. Her blood culture was positive for Staphylococcus aureus. Her echocardiogram revealed small left ventricle with ejection fraction of 65%, flattened interventricular septum, normal left atrium (LA), bowing of the interatrial septum to the LA, right atrial (RA) and RV enlargement with severe hypokinesia of RV (Fig. 1). Estimated RV systolic pressure was 75 mmHg. She also had TEE to look for any vegetation. There was no vegetation. Her echo examination 8 months ago was normal. High resolution Chest computerized tomography (CT) revealed normal lungs and CT angiogram revealed no evidence of pulmonary embolism. The patient underwent left and right heart catheterization10 days later. Coronary arteries and LV function were normal. RA pressure 6 mmHg, RV, 36/11 mmHg, pulmonary artery 36/11 mmHg, mean 24 mmHg, indicating significant improvement of pulmonary hypertension. Echo on the next day revealed markedly improved RA and RV dilatation (Fig. 1). Patient had a full recovery on discharge.

## Discussion

Early circulatory response to sepsis and septic shock is characterized by vasodilation and increased vascular permeability, which leads to a marked reduction in intravascular filling pressures. As a result, LV ejection fraction (LVEF) often decreases during sepsis [1]. Unlike the systemic circulation, resistance in the pulmonary vasculature is often increased in sepsis, especially in the presence of acute lung injury [2]. Acute lung injury leads to hypoxic pulmonary vasoconstriction, and this pulmonary pressor response is enhanced by hypercarbia or acidosis. Decreased production of nitric oxide, an endogenous vasodilator, likely contributes to increased PVR and pulmonary hypertension in sepsis [3]. Coagulation abnormalities are also common in the pulmonary vascular bed during sepsis and acute lung injury and may lead to pulmonary arterial thrombosis that further raises PVR. The RV is poorly prepared to compensate for acute elevations in afterload. Aggressive fluid resuscitation and frequent CVP monitoring are often necessary to ensure adequate right-sided filling pressures. Although volume expansion should be the initial treatment for RV dysfunction in septic shock, a threshold exists beyond which further fluid resuscitation is detrimental. The presence of peripheral edema or jugular venous distension may indicate elevated right-sided pressures but may not necessarily indicate RV failure or even adequate RV filling. A prudent approach is to assess RV function in patients who continue to show signs of inadequate cardiac output after initial volume resuscitation [4]. If echocardiography reveals decreased RV contractility, fluid therapy should be guided based on the CVP measurement. Increases in CVP, without increase in cardiac output, may impede LV filling which could in turn worsen the RV dysfunction. On the other hand, if cardiac output increases with further elevation of CVP, maintenance of higher right-sided filling pressures may be warranted. Nitric oxide improves PVR and decreases RV afterload and may be useful therapy in bridging patients with acute RV failure in septic shock [5].

### Conclusion

Right ventricular dysfunction is common in sepsis and septic shock because of decreased myocardial contractility and elevated PVR despite a concomitant decrease in systemic vascular resistance. Aggressive volume replacement may be vital in maintaining RV function, but excess hydration can cause RV dilation, decreased LV diastolic filling, and reduced cardiac output. The mainstay of treatment for acute right heart dysfunction in the setting of sepsis should concentrate on fluid repletion, monitoring for signs of RV overload, and correcting reversible causes of elevated PVR, such as hypoxia, acidosis, and lung hyperinflation.
